# Pharmacological prevention of surgery-accelerated metastasis in an animal model of osteosarcoma

**DOI:** 10.1186/s12967-020-02348-2

**Published:** 2020-04-30

**Authors:** Michelle P. Kallis, Caroline Maloney, Brandon Blank, Samuel Z. Soffer, Marc Symons, Bettie M. Steinberg

**Affiliations:** 1 The Elmezzi Graduate School of Molecular Medicine, Northwell Health, Manhasset, NY USA; 2 The Institute of Molecular Medicine, The Feinstein Institutes for Medical Research, 350 Community Drive, Manhasset, NY 11030 USA; 3 Department of Surgery, Donald and Barbara Zucker School of Medicine at Hofstra/Northwell, New Hyde Park, NY USA; 4 Department of Molecular Medicine, Donald and Barbara Zucker School of Medicine at Hofstra/Northwell, Manhasset, NY USA; 5 Donald and Barbara Zucker School of Medicine at Hofstra/Northwell, Manhasset, NY USA

**Keywords:** Surgery-accelerated metastasis, Osteosarcoma, Metastasis-associated macrophages, Gefitinib

## Abstract

**Background:**

Osteosarcoma is a highly metastatic primary bone tumor that predominantly affects adolescents and young adults. A mainstay of treatment in osteosarcoma is removal of the primary tumor. However, surgical excision itself has been implicated in promoting tumor growth and metastasis, an effect known as surgery-accelerated metastasis. The underlying mechanisms contributing to surgery-accelerated metastasis remain poorly understood, but pro-tumorigenic alterations in macrophage function have been implicated.

**Methods:**

The K7M2-BALB/c syngeneic murine model of osteosarcoma was used to study the effect of surgery on metastasis, macrophage phenotype, and overall survival. Pharmacological prevention of surgery-accelerated metastasis was examined utilizing gefitinib, a receptor interacting protein kinase 2 inhibitor previously shown to promote anti-tumor macrophage phenotype.

**Results:**

Surgical excision of the primary tumor resulted in increases in lung metastatic surface nodules, overall metastatic burden and number of micrometastatic foci. This post-surgical metastatic enhancement was associated with a shift in macrophage phenotype within the lung to a more pro-tumor state. Treatment with gefitinib prevented tumor-supportive alterations in macrophage phenotype and resulted in reduced metastasis. Removal of the primary tumor coupled with gefitinib treatment resulted in enhanced median and overall survival.

**Conclusions:**

Surgery-accelerated metastasis is mediated in part through tumor supportive alterations in macrophage phenotype. Targeted pharmacologic therapies that prevent pro-tumor changes in macrophage phenotype could be utilized perioperatively to mitigate surgery-accelerated metastasis and improve the therapeutic benefits of surgery.

## Background

Osteosarcoma (OS) is the most common malignant bone tumor of adolescence, and the third most common cancer in adolescence overall. It is a highly metastatic disease, with approximately 20% of patients already presenting with lung metastases at the time of diagnosis. Most of the remaining patients without metastasis at diagnosis are thought to have subclinical micrometastases, and 30–40% of those patients will go on to develop overt metastatic disease [1]. The presence of metastatic disease severely reduces the overall survival of patients, from nearly 80% to less than 20%. Despite advancements in chemotherapeutic and surgical approaches in the treatment of OS, the overall survival for patients with metastatic OS has not improved in three decades [2, 3].

As is the case with most solid tumors, surgical resection of the primary tumor in OS remains a mainstay of treatment [3, 4]. However, there is increasing evidence that surgical interventions may result in physiologic processes that serve to promote local recurrence and metastatic disease [4–6]. The underlying mechanisms that contribute to surgically-accelerated metastasis remain unclear, but it has been repeatedly demonstrated that surgery results in a generalized immunosuppressive state and alters cell-mediated immune function [6–9]. Although the functions of multiple immune cell types have been shown to be modulated following surgery, macrophage function specifically has been shown to be altered [8–11].

It has also been demonstrated that tumor-associated macrophages (TAMs), macrophages present within the microenvironment of solid tumors, play a critical role in the pathogenesis of OS through regulation of the tumor microenvironment [12, 13]. While a multitude of macrophage subtypes may exist within the tumor microenvironment, macrophages exhibiting tumor supportive functions predominate in most cancers. These TAMs secrete pro-angiogenic factors, inhibit NK and T-cell function, and facilitate tumor cell extravasation and invasion by promoting stromal remodeling [14–16]. While many studies have demonstrated the role of TAMs in tumor progression in the primary tumor, less is known about the macrophages present at the metastatic site, metastasis-associated macrophages (MAMs), which promote later stages of metastasis. MAMs appear to promote metastatic tumor development in a manner similar to TAMs by initiating angiogenesis, inhibiting anti-tumor immune responses, and promoting matrix remodeling making MAMs potential targets for novel therapeutic intervention to inhibit metastatic development [17].

Previous studies in both melanoma and breast cancer have demonstrated that surgical wounding results in systemic increases in the myeloid precursors to TAMs and MAMs, increases in macrophage density within metastatic nodules, pro-tumor polarization in TAMs, and increases in tumor progression and metastatic outgrowth [11, 18]. Those studies also demonstrate that the deleterious effects of surgery could be abrogated through either macrophage depletion or re-education [11, 18]. Much less is known about surgically-accelerated metastasis in OS, and there are no therapies available that specifically target this process in OS or any other tumor type.

In this current study, we have used the K7M2-BALB/c syngeneic orthotopic murine model of metastatic OS to examine the effects of surgery on metastatic growth in OS, investigate the role that macrophages play in post-surgical metastatic enhancement, and ascertain whether treatment of the mice with gefitinib, an already FDA approved medication which has been shown to alter macrophage function through an off-target inhibition of receptor interacting protein kinase 2 (RIPK2) [19], can prevent the metastatic enhancement induced by surgery.

## Methods

### Cell lines and cell culture

The K7M2 (ATCC CRL-2836) murine cell line was purchased from the American Type Culture Collection. Cells are cultured in DMEM (Gibco) supplemented with 10% heat-inactivated Fetal Clone II (HyClone Laboratories), 2 mM l-glutamine (Gibco), and 1% penicillin/streptomycin (HyClone). All cells were used between passages 4 and 12, and all cell lines were confirmed free of mycoplasma using ATCC universal mycoplasma detection kit. Date of last mycoplasma testing was 11/26/19.

### Animal studies

All animal procedures were approved by, and in accordance with, the ethical standards of the Institutional Animal Care and Use Committee (IACUC protocol #2015-056) of The Feinstein Institutes. Female BALB/c mice (The Jackson Laboratory), 4 to 5 weeks of age, date of birth ± 3 days, were housed under specific pathogen-free conditions with five mice per cage in a 12-h light/dark cycle with ad libitum access to food and water.

### Intratibial injections

K7M2 OS cells were trypsinized, washed with PBS, and viability confirmed at greater than 90% as assessed by trypan blue exclusion. Mice were anesthetized with 2–3% inhaled isoflurane, received pre-emptive analgesia with 0.1 mg/kg of buprenorphine via SQ injection, and 10 μL of the cell suspension containing 1 × 10^5^–3 × 10^5^ cells was injected slowly into the anterior intercondylar area of the tibia of the left hind limb using a 28 g syringe (Hamilton), as previously described [20]. Mice continued to receive 0.1 mg/kg of buprenorphine SQ for post-operative analgesia BID for the first post-operative day.

### Limb resection surgery

One week after intratibial tumor injections, mice underwent amputation of the primary tumor-bearing limb. Mice were anesthetized with 2–3% isoflurane and received pre-emptive analgesia at the start of the procedure with buprenorphine as described above. Using sterile surgical scissors a circumferential skin incision was made over the proximal femur to expose the femoral vessels. The femoral neurovascular pedicle was ligated using a 4-0 Vicryl suture (Ethicon). Musculature proximal to the tumor margin was sharply transected to allow for visualization of the femur. Resection of the limb was done at the level of the distal femur, proximal to the tibial tumor, so as to minimize physical manipulation of the primary tumor during resection. In animals where the tumor-bearing limb was left in situ, and the non-tumor bearing contralateral limb was resected, resection occurred at this same anatomical location. A single interrupted 4-0 Vicryl stitch was used to close the overlying muscle over the femoral stump. Superficial skin was closed with 9 mm stainless steel wound clips (MikRon Precision Inc.), clips were then removed on post-operative day 10. Animals received post-operative analgesia with buprenorphine (0.1 mg/kg) on post-operative days 1–3. At 3 weeks post-operatively, 4 weeks following tumor inoculation, mice were euthanized and lungs were harvested for subsequent analysis.

### In vivo drug treatment

Gefitinib (Biotang, Inc) was formulated into chow at 100 mg/kg/day (Research Diets) [21]. Mice treated with gefitinib developed a mild rash, a known side effect of gefitinib, and no significant weight loss, indicating that mice were eating appropriate amounts of chow. Treatment with gefitinib began 5 days after tumor inoculation (2 days prior to surgical resection). Treatment with gefitinib was continued for either 1 week or until sacrifice, as indicated within the text and figure legends.

### Assessment of pulmonary metastasis

Mice were euthanized by CO_2_ asphyxiation, lungs were dissected out of the thorax, and fixed with 10% neutral-buffered formalin. The number of gross metastatic nodules on the surface of the intact lung was counted by two independent researchers. Lungs were cut into multiple portions and paraffin imbedded so that 5 μm thick longitudinal sections from each paraffin block would contain all portions of the lung. Sections were stained by routine hematoxylin and eosin methods and examined on an Axiovert 200M inverted microscope (Carl Zeiss AG) with AxioVision software (Version 4.8.2.0; Carl Zeiss AG) to visualize 50–100% of the lung tissue per microscopic field. Metastatic foci were defined as all metastases ≥ 4 cells. Average focus size was calculated by measuring the surface area of all foci in each section, as previously defined, using ImageJ software (Version 1.47; National Institutes of Health) and dividing by the total number of foci within each section. Two histological sections/paraffin block were examined and results averaged. Metastatic burden was calculated by measuring the total surface area of metastatic foci in each section, also using ImageJ software, and dividing by the total surface area of lung tissue in each section. Two independent researchers utilized these methods to measure average focus size and metastatic burden in a blinded fashion with high concordance (r2 = 0.98; 95% confidence interval [CI] 0.95–0.99).

### Flow cytometry

Lung tissue was roughly cut with surgical scissors and placed in gentleMACS™ C Tubes (Miltenyi Biotec) with digestion media comprised of 100 U/mL collagenase type 1 (Gibco) and 20 U/mL DNase (Invitrogen). Tubes were placed into gentleMACS™ dissociator and a pre-programmed lung digestion protocol was run for 30 min at 37 °C. The resultant cell suspension was passed through a 70-μm filter (BD Falcon) and proteases quenched with DMEM + 10% FCS. Single cells were washed in PBS containing 1% FCS, red blood cells lysed and cells incubated with CD16–CD32 antibodies (Fc block; 2.4G2) to block Fc receptors prior to staining. Cells were incubated with Zombie Aqua (Biolegend) live-dead staining for 30 min according to manufacturer’s instructions, using heat-killed cells as a positive control. For surface staining, cells were incubated with fluorescently labeled antibodies from BioLegend unless otherwise specified: mouse FITC CD45 (30-F11), PE/Cy7 F4/80 (BM8), PerCPCy5.5 CD206 (C068C2), and APC I^A^/I^E^ (Major Histocompatibility Complex Class II (MHCII)) (M5/114.15.2). After staining, cells were fixed with 1% paraformaldehyde and stored at 4 °C until data acquisition on an LSR Fortessa (BD Biosciences). Post-acquisition analysis was performed with FlowJo software (V. 10.0.7r2, FlowJo LLC). Cells were first gated based on forward- and side-scatter properties, followed by gating on CD45^+^live cells before identifying the individual population of interest.

### Statistical analysis

**Fig. 1:**
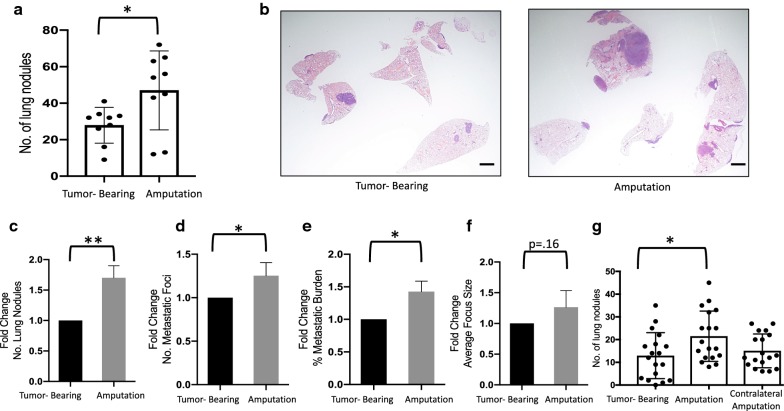
Surgical excision enhances metastatic progression of pre-existing micrometastases. **a**, **b** BALB/c mice were implanted with 3 × 10^5^ cells in the left tibia and 1 week after implantation were randomly assigned to undergo either removal of their primary tumor via amputation of the tumor bearing limb (amputation, n = 10) or no surgery (tumor-bearing, n = 10). Mice were sacrificed 4 weeks after implantation (3 weeks post-surgery). **a** Number of nodules on surface of lungs. Scatter plots show individual mice. **b** Micrographs of mouse lungs 4 weeks after implantation of tumor cells (scale bar = 500 µm). **c**–**f** Combined data of three individual experiments (n = 10–15 per group per experiment). Mice were implanted with 1 × 10^5^–3 × 10^5^ cells; experimental design as in (**a**). Data normalized to tumor-bearing average for each experiment and expressed as fold change relative to tumor-bearing average. **c** Gross nodules on surface of lung. **d** Number of micrometastatic foci identified on histologic analysis. Single focus defined as ≥ 4 tumor cells. **e** Metastatic burden, calculated by measuring the total area of metastatic foci divided by the total area of the lung section. **f** Average focus size, calculated by measuring total area of metastatic foci divided by total number of foci. Data was compared with two-tailed Student’s t-test, *p < 0.05, **p < 0.01. **g** Mice were implanted as in (**a**) and randomly assigned to undergo either amputation of the tumor bearing limb (amputation, n = 18), amputation of the contralateral, non-tumor bearing-limb (n = 18), or no surgery (tumor-bearing, n = 18). Mice were sacrificed 4 weeks after implantation (3 weeks post-surgery). Data is the aggregate of two independent experiments. Data was compared with one-way ANOVA with post hoc Dunnett’s multiple comparisons test *p < 0.05. All graphs show mean ± standard deviation

**Fig. 2:**
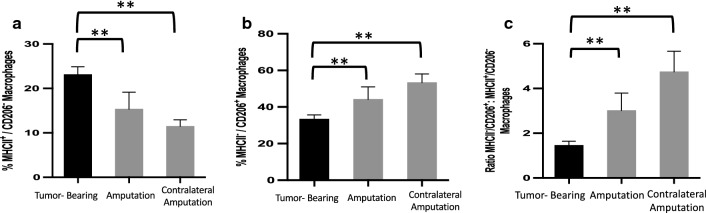
Macrophages within the metastatic niche are shifted to pro-tumor state in the acute post-operative period. **a**–**c** Mice were treated as in Fig. 1g, then euthanized 48 h after surgical interventions (9 days after tumor implantation) and lungs processed into single cell suspensions. Flow cytometric analysis of lungs from tumor-bearing mice (n = 10), mice that underwent surgical excision of their tumor-bearing limb (amputation, n = 10), or mice that underwent surgical excision of their contralateral, non-tumor bearing-limb (n = 10) were compared via flow cytometric analysis. After gating for viability, cells were gated for size, singlets, and CD45, F4/80 double positivity, to define the general macrophage population. CD45^+^/F4/80^+^ cells were analyzed for the percentages of surface markers CD206 and MHCII. **a** Percentage of MHCII^+^/CD206^−^ macrophages (anti-tumor macrophage population). **b** Percentage of/MHCII^−^/CD206^+^ macrophages (pro-tumor macrophage population). **c** Pro-tumor macrophages: anti-tumor macrophage ratio. Bars represent mean ± standard deviation compared by one-way ANOVA with post hoc Dunnett’s multiple comparisons test **p < 0.01

**Fig. 3:**
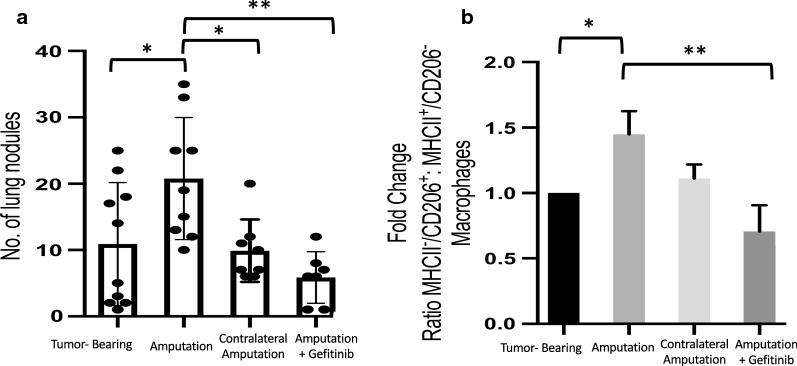
Gefitinib treatment prevents surgery-accelerated metastasis and reverses post-surgical pro-tumor macrophages polarization. **a** BALB/c mice were implanted with 3 × 10^5^ cells in the left tibia and 1 week after tumor implantation mice underwent surgical excision of primary tumor bearing limb or amputation of the contralateral limb as in Fig. 1g. Mice assigned to gefitinib treatment were started on gefitinib-impregnated chow (100 mg/kg/day) 2 days prior to surgical excision (5 days after tumor implantation). Gefitinib treatment was continued for 1 week following surgery, and mice were sacrificed 3 weeks after surgery (4 weeks after tumor implantation). **a** Number of gross nodules on surface of lungs (n = 7–10 mice per group). **b** Mice were treated as in Fig. 1. Lungs were made into single-cell suspensions and analyzed via flow cytometric analysis with gating strategy as in Fig. 2. Combined data from three independent experiments (n = 4–6 mice per group per experiment). Pro-tumor macrophage: anti-tumor macrophage ratio. Scatter plots show individual mice, all graphs displayed with means ± standard deviations. Data analyzed using one-way ANOVA with post hoc Tukey’s multiple comparison test,*p < 0.05, **p < 0.01

**Fig. 4:**
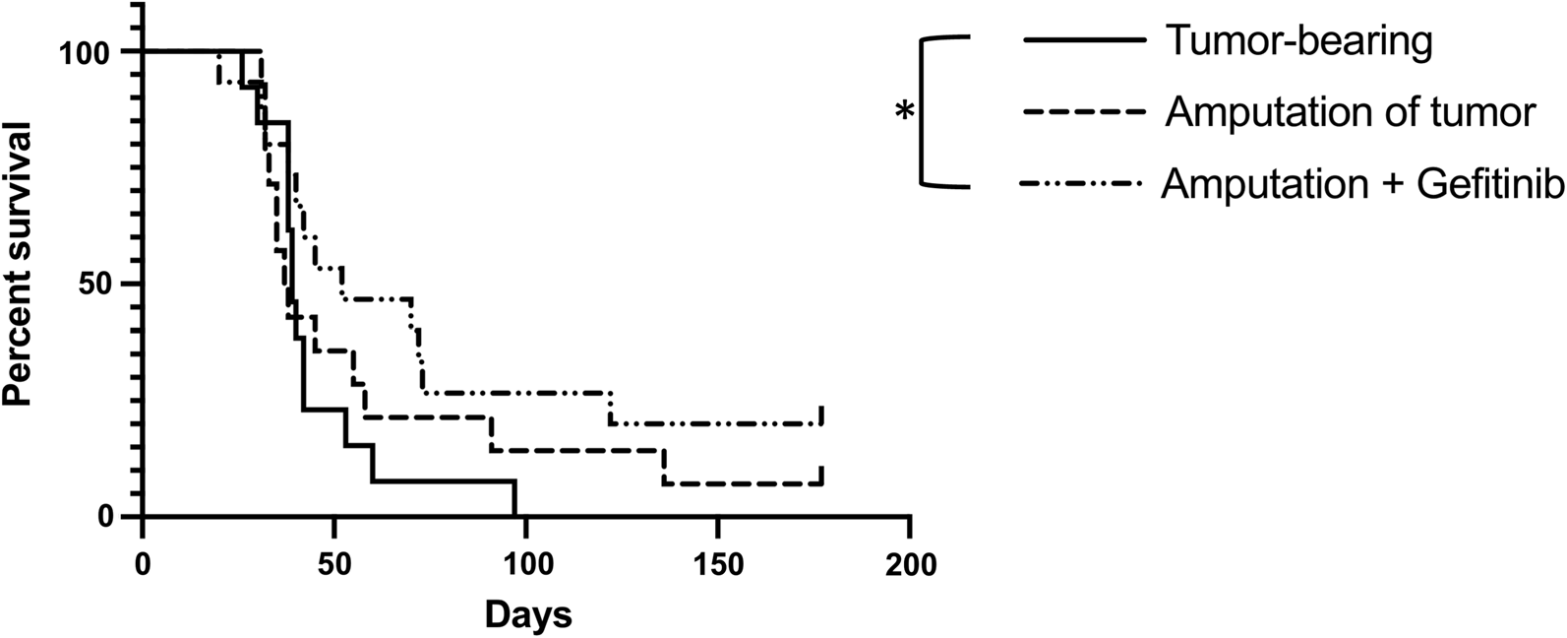
Surgery coupled with systemic treatment to reeducate macrophages yields survival benefits BALB/c mice were implanted with 3 × 10^5^ cells in the left tibia (n = 13–15 mice/group). One week after tumor implantation mice assigned to surgical groups underwent surgical excision of primary tumor bearing limb as in Fig. 1. Mice assigned to gefitinib treatment were started on gefitinib-impregnated chow (100 mg/kg/day) 2 days prior to surgical excision (5 days after tumor implantation). Gefitinib treatment was continued for the duration of the experiment. Mice were sacrificed upon meeting euthanasia criteria as previously described. Data analyzed using Log-rank (Mantel–Cox) test,*p < 0.05

We determined that ten mice per group would be sufficient to measure a difference in means of 25% with 90% power. In our extensive characterization of the K7M2-BALB/c syngeneic model of OS, we have demonstrated that our metastatic efficiency is 90%, and that we are directly seeding the lungs with tumor cells via an intraosseous injection [20]. Therefore, upon histological analysis of lung sections if no single metastatic focus could be identified, the specimen was classified as a technical implantation error and was excluded from further analysis. As a result, in experiments described in Figs. 1, 2 and 3 a total of 8 mice were excluded from analysis, 3 from tumor-bearing group, 3 from amputation group, and 2 from amputation with gefitinib group and a total of 2 mice were excluded from the survival experiment in Fig. 4, one mouse from the tumor-bearing group and one mouse from the amputation group.

Unpaired, two-tailed *t* tests or ordinary one-way analysis of variance (ANOVA) with post hoc multiple comparisons test were utilized as indicated in figure legends. Chi-square tests were used for proportional analysis of animal survival. Survival analysis was conducted with Log-rank test and depicted using Kaplan–Meier survival plot. All bar graphs depict data as mean ± standard deviation. Figures were created and statistical analyses were performed using Prism (V. 8.1.0, GraphPad Software Inc.); p-value < 0.05 was considered to be statistically significant.

## Results

### Surgical excision of the primary tumor enhances growth of pre-existing pulmonary micrometastases

We first investigated whether surgical removal of the primary tumor would affect metastatic growth in the K7M2-BALB/c syngeneic model of OS that we have extensively characterized [20]. To test this, 1week following tumor inoculation, the primary tumor-bearing limb was amputated in the surgical group. Based upon data from our previous study on the metastatic kinetics of this model, we know that micrometastases are present within in the murine lung at this timepoint [20].

At 3 weeks after surgical excision, all mice were euthanized and the number of gross metastatic nodules present on the surface of the lung was quantified. There was a 68% increase in the average number of gross metastatic nodules present on the surface of the lungs in the surgical group, compared to non-operative controls (Fig. 1a; p = 0.028). The histologic appearance of lungs of mice from each group is represented in Fig. 1b.

To examine the consistency of these results, we performed three independent experiments, varying the number of cells injected, with very similar results (Fig. 1c). In addition to gross metastatic nodules, surgery’s effects on metastatic foci and metastatic burden were also examined. Following surgical resection, we observed significant increases in the number of gross nodules, the number of metastatic foci, and overall metastatic burden in the lung (Fig. 1c–e). Although there was also a trend toward an increase in average size of metastatic foci, this did not achieve statistical significance due to larger variability of this parameter (Fig. 1f). However, the effect-size of surgery on the average size of foci was similar to that of the number of metastatic foci, suggesting that surgery is also likely to increase the average size of each focus, but that our study was under-powered to examine this parameter.

We next asked whether the increase in metastasis following surgical resection was related to removal of the primary tumor or whether surgical wounding itself provokes metastatic outgrowth. To examine this, we repeated the experiment shown in Fig. 1a, but included an additional group where the primary tumor bearing-limb was left in place and the contralateral, non-tumor bearing-limb, was resected (Fig. 1g). Surgical resection of the primary tumor-bearing limb increased the mean number of gross metastatic nodules 66% compared to non-operated controls (p = 0.02). Surprisingly, surgical resection of the contralateral non-tumor bearing-limb did not produce the same increase in gross metastatic nodules suggesting that the effect of surgery-accelerated metastasis in our model is provoked by removal of the primary tumor, and is not secondary to surgical stress alone.

### Acute surgical stress shifts macrophage polarization toward a pro-tumor state within the metastatic niche

As previous studies in other cancer models demonstrate, surgery can increase the predominance of macrophages systemically and within the primary tumor [10, 11, 18]. We therefore sought to examine the effect of surgical wounding, on macrophages within the metastatic niche in OS. To investigate this, we used flow cytometric analysis to analyze macrophage expression of major histocompatibility complex class II (MHCII) and mannose receptor (CD206) on pulmonary macrophages isolated from tumor-bearing mice, mice that underwent surgical resection of their tumor-bearing limb, or mice that underwent amputation of their contralateral limb, leaving their primary tumor in place. MHCII molecules allow macrophages and other antigen-presenting cells to activate T-cells and initiate the immune response. As a result, macrophages expressing high levels of MHCII have been shown to support anti-tumor functions [22]. Conversely, macrophages expressing low levels of MHCII and high levels of CD206 have been shown to be primarily tumor-supportive in nature with high production of IL-10 and poor antigen-presenting capabilities. Functionally, these MHCII^−^/CD206^+^ macrophages act as tumor-supportive macrophages through inhibition of anti-tumor immunity, as well as through promotion of vessel formation by secretion of angiogenic factors and initiation of metastasis through matrix remodeling [22–26].

As monocyte precursors of macrophages are systemically mobilized and macrophages within tissue begin to rapidly alter their phenotype in response to spatiotemporal cues as soon as 24 h after surgical wounding [27], we first examined changes in macrophage expression 48 h after surgical intervention. While the overall number of macrophages within the lung did not change, at this acute post-operative time point there was a significant reduction in the percentage of MHCII^+^/CD206^−^ macrophages (Fig. 2a) and an increase in the percentage of MHCII^−^/CD206^+^ macrophages (Fig. 2b), resulting in a marked increase in the ratio of MHCII^−^/CD206^+^ to MHCII^+^/CD206^−^ macrophages (Fig. 2c) in both surgical groups, regardless of whether the primary tumor was removed or left in situ. Thus, these data demonstrate a significant shift in the macrophage phenotype within the lung to a more pro-tumor state in the acute post-operative period simply due to surgical stress. Interestingly, this effect appears to be more pronounced within macrophages from the contralateral group, but the implication of this is unclear.

### Pro-tumor macrophage phenotype persists following resection of primary tumor. Gefitinib reverses the effects of surgical resection of primary tumor on metastasis and macrophage polarization

Given that contralateral limb amputation produced the same acute tumor-supportive shift in pulmonary macrophage phenotype as primary tumor resection, we asked whether this change in macrophage polarization persisted 3 weeks after surgery, the time point at which we had seen a significant increase in metastatic nodules following primary tumor resection, but not after contralateral amputation. Flow cytometric analysis of pulmonary macrophages demonstrated that the shift in macrophage phenotype toward a pro-tumor state persisted at 3 weeks after surgical removal of the primary tumor (Fig. 3b). In contrast, the ratio of pro-tumor to anti-tumor macrophages within the contralateral amputation group was down to nearly that of non-operated controls. These data suggest that it is the persistence of the pro-tumor macrophage phenotype that leads to enhanced metastatic outgrowth following surgical resection of the primary tumor, and that this persistence of pro-tumor macrophage phenotype is facilitated by removal of the primary tumor. Additionally, we previously reported that gefitinib, generally considered an EGFR inhibitor, is able to reduce metastasis in a non-surgical model of OS through alteration of macrophage phenotype by inhibiting macrophage RIPK2 [19]. We therefore asked whether peri-operative gefitinib treatment could prevent the enhanced metastasis and pro-tumor polarization of macrophages following surgical resection of the primary tumor. To test this, gefitinib was administered orally via drug-impregnated chow initiated on day 5 following tumor inoculation, and mice underwent surgery 2 days later. Gefitinib was continued for 1 week, and mice were sacrificed at 3 weeks post-surgery. Treatment with oral gefitinib within the perioperative period alone successfully prevented the enhanced metastatic effect of surgical removal of the primary tumor and reduced the number of metastatic pulmonary nodules below that of non-operative controls (Fig. 3a). Moreover, peri-operative gefitinib treatment was able to prevent the induction or persistence of the pro-tumor macrophage phenotype within the metastatic niche (Fig. 3b).

### Gefitinib enhances overall survival in the K7M2-BALB/c syngeneic model of OS

Surgical excision of the primary tumor is standard treatment for patients with OS. As surgical resection of the primary tumor-bearing limb increased gross metastatic nodules as well as metastatic foci and burden in our murine model, we next investigated what impact this post-surgical metastatic enhancement would have on survival, and whether gefitinib would increase survival if used as an adjunct to surgical treatment. To test this, mice were again inoculated via intratibial injection with OS cells. The gefitinib group began oral gefitinib treatment 5 days after tumor injection, which was continued as a maintenance therapy for the duration of the experiment. Surgical resection of the tumor-bearing limb was completed on day 7 following tumor inoculation, as done in the prior experiments. Mice were sacrificed upon meeting euthanasia criteria, which were defined as weight loss greater than 20% of weight at start of experiment or a score of 7 or higher as defined by the mouse grimace scale [28].

Median survival of mice undergoing surgical resection was slightly reduced (by 1.5 days) compared to non-operative mice (37.5 vs 39 days, respectively), although this difference did not reach statistical significance (p = 0.28, Fig. 4). This decrease in survival due to metastasis may be an underestimate given that some tumor-bearing mice may have succumbed secondarily to effects of the primary tumor and not solely due to their metastatic burden. This is evidenced by mice within the tumor-bearing group succumbing with less metastatic burden compared to both surgical resection alone and surgical resection with gefitinib treatment (37.4% vs 55.65% and 59.2%, respectively; p < 0.05, Additional file 1). However, no mice within the tumor-bearing group achieved long-term survival, defined as animals living to day 100 or greater, whereas 14% of mice within the surgical resection group survived to this time point (Fig. 4). Taken together, these data suggest that removal of the primary tumor does provide some benefit despite the effects of surgery on metastases.

Treatment of mice undergoing surgical resection with gefitinib increased median survival to 52 days, representing a 33.4% increase in lifespan relative to tumor-bearing mice, and an overall significant increase in survival (p < 0.05, Fig. 4). Surgical resection combined with gefitinib treatment also increased the proportion of long term survivors from 0% in the tumor-bearing group to 26.7% (p < 0.05, Fig. 4). Thus, gefitinib clearly has therapeutic value as a pharmacological adjunct to surgery in this model of osteosarcoma.

## Discussion

In this study we describe a robust model of surgery-accelerated metastasis in OS, allowing for the mechanistic analysis of this effect. We demonstrate that surgical excision of the primary tumor-bearing limb resulted in significant increases in the number of gross metastatic nodules, the number of micrometastatic foci, overall metastatic burden, and effect-size consistent increases in the average size of each metastatic focus. Surprisingly, subjecting animals to equivalent surgical stress through amputation of the contralateral non-tumor bearing-limb, leaving the primary tumor in place, did not produce the same enhancement of metastasis. This strongly indicates that surgery-accelerated metastasis is mediated, at least in part, by the absence of the tumor and not just factors intrinsic to surgical wounding. Additionally, we implicate macrophages as key mediators in this effect by demonstrating that surgical excision of the primary tumor produces persistent changes in macrophage phenotype within the metastatic niche to that of a more pro-tumor state. Finally, we provide evidence that utilizing gefitinib as a peri-operative adjunct to surgery can effectively mitigate the harmful effects of surgical excision of the primary tumor by preventing tumor supportive phenotypic changes in macrophages, decreasing metastatic burden, and enhancing survival.

Although surgery-accelerated metastasis has been repeatedly described, the underlying mechanisms that contribute to this effect remain poorly understood, and as a result there is a paucity of targeted treatments. Advancements in the study of surgery-accelerated metastasis have been limited in significant part due to the difficulty of developing experimental animal systems that can accurately recapitulate the human condition. In the model of surgery-accelerated metastasis in OS used in this study, we directly seed the lungs with micrometastatic foci through an intraosseous injection producing both a primary tumor that can then be manipulated, as well as pulmonary micrometastases that can be subsequently analyzed [20]. Clinically, we know that the majority of OS patients have micrometastatic pulmonary disease at the time of primary tumor resection, thus by conducting surgery 1 week after micrometastases are present in the lung we are able to study the effects of surgery on the later steps of metastatic progression, recapitulating this clinical scenario.

We also examined the effects of surgery on survival in this K7M2-BALB/c model of OS. We found that even though surgical excision of the primary tumor may produce a small reduction in median survival compared to tumor-bearing controls, this surgical intervention did result in a small number of long-term survivors compared to the tumor bearing group (2 animals vs 0 animals, respectively). These data suggest that despite the pro-metastatic effects of surgery, there may be benefits to removal of the primary tumor, possibly caused by morbidity of the growing primary tumor itself. Consistent with this are the findings of the study by Rashid et al. [29], which demonstrated that although removal of the primary tumor in a murine model of metastatic breast cancer resulted in accelerated growth of metastatic lesions, overall survival was improved by primary tumor resection. Furthermore, the authors demonstrate that when primary tumor resection decreased overall tumor burden substantially, accelerated growth of metastatic lesions did not increase overall tumor burden compared to the no-surgery group and survival was subsequently improved, which was not the case when primary tumor resection did not significantly reduce overall tumor burden [29]. Our observations are also consistent with the findings of Simpson-Herren et al. [30] in a mouse model of Lewis lung cancer, where surgical excision of the primary tumor resulted in a significantly increased growth rate of lung metastasis, resulting in a small, but consistent, decrease in median survival. Furthermore, in a study by Dillekås et al. examining the recurrence patterns of women with breast cancer after mastectomy over a 30 year period, it was found that while breast surgery was clearly an independent stimulating event for the growth of metastases and increased relapse, it did not result in worse long-term disease-free survival [31]. Taken together these data indicate that despite some of the harmful effects of surgery, the benefit of surgical procedures in diagnosis, treatment, and cure of oncologic disease is generally considered to be without argument [4]. The importance of demonstrating that there are in fact unintended tumor-promoting effects of surgery is that we can now focus on understanding the mechanisms of these effects in order to produce targeted therapies to further augment the benefits of surgery.

While there are many hypotheses as to the underlying mechanisms contributing to surgery-accelerated metastasis, one of the most pervasive is that surgical wounding generates a permissive tumor environment through alterations in immune function [4, 6–11, 18, 32]. Increased macrophage infiltration of primary tumors outside the context of surgery has been shown to portend a poor prognosis [33, 34]. Functionally, TAMs appear to be predominantly alternatively activated macrophages exhibiting immunosuppressive and pro-tumorigenic functions. However, conceptualizing macrophages strictly as either pro-tumor or anti-tumor types is an over simplification of their complex biology and plastic nature. While generally TAMs appear to be tumor supportive by secreting pro-angiogenic, pro-growth, and immunosuppressive factors, there are in actuality a multitude of different macrophage types associated with the tumor.

Few studies have looked at changes in TAM populations after surgery. Krall et al. [11] demonstrated in a mouse model of breast cancer that surgical wounding results in a systemic upregulation of macrophages, increasing their availability to be recruited into tumors. Furthermore, that study demonstrated that the macrophages that were recruited into tumors following surgical wounding showed increased expression of CD206 on their surface, a marker of pro-tumor macrophage function [11]. Similarly, Tham et al. found that following resection of primary melanoma in a mouse model there is increased infiltration of macrophages both within primary tumor recurrences and within metastases, and that this macrophage infiltration is associated with increased proliferation of tumor cells. However, the phenotype of the macrophage infiltrate within the metastases was not examined in their study [18]. Interestingly, Maloney et al. [19] also reported that the proliferative capacity of K7M2 OS cells was enhanced when co-cultured with bone marrow-derived macrophages in an in vitro assay. Although the exact phenotype of the macrophages used in that assay was not characterized, those macrophages were grown in media containing M-CSF, a known driver of a pro-tumor macrophage phenotype [19]. In this current study we have shown a trend toward an increase in the average size of metastatic foci following surgical resection, which was associated with a shift in macrophages to a pro-tumor phenotype, suggesting that pro-tumor macrophages can enhance proliferation in OS as well.

To our knowledge this is the first report demonstrating that surgical resection of the primary tumor induces a sustained shift in macrophage phenotype within the metastatic niche to a pro-tumor state, correlating with enhanced metastatic outgrowth in OS. The focus of our study was specifically on the alterations to macrophage function within the metastatic niche following surgical resection, and how this contributes to surgery-accelerated metastasis. However, we cannot exclude the role of other post-surgical physiologic changes in metastatic enhancement. Some of these proposed mechanisms include release of immunologic and neuroendocrine factors such as prostaglandins, catecholamines, and glucocorticoids, creation of a systemic pro-angiogenic environment, and release of wound-healing related growth factors [5, 6, 32]. We also cannot exclude the effect of changes to other immune cell types within the metastatic niche such as T-cells or NK cells, or the effect of changes to myeloid cells in circulation after surgery. Examining how these other immune cell types change following surgery and potentially contribute to surgery-accelerated metastasis is a natural continuation of our work and will be the focus of future studies. It is most likely that multiple mechanisms act in conjunction with one another to ultimately support tumor recurrence and metastatic outgrowth after surgical intervention. However, pro-tumor macrophages may contribute to the other proposed mechanisms as well, as pro-tumor macrophages themselves secrete angiogenic factors, growth factors, matrix-remodeling proteins, cytokines, and chemokines [4, 14, 32]. Thus, by investigating post-surgical changes in macrophages we may garner a better understanding as to how the mechanisms underlying surgery-accelerated metastasis may be linked, to which this report contributes. Currently, we are engaged in further studies to correlate the post-surgical changes in macrophage phenotypic markers identified in this study with functional changes in these macrophages by using PCR analysis. We plan to examine macrophage gene expression changes following surgery in genes specifically related to cancer and immunity crosstalk, including genes related to angiogenesis, matrix-remolding, and growth factor production. This will be the work of a future study.

The design of the surgical model used in this study was such that the entire tumor bearing limb of the mouse was amputated, as limb sparing surgery in a mouse model is technically prohibitive. This model was chosen to mimic as closely as possible the clinical situation of total resection of the primary tumor with pre-existing pulmonary micrometastases. Despite its benefits, the orthotopic model of osteosarcoma used in this study has some important limitations that should be noted. As discussed previously by other authors, injection of a tumor cell suspension into the murine tibia is a technically difficult process due to its curved anatomy and the limited volume of the tibial marrow cavity [35, 36]. These technical limitations of the model, along with intrinsic variations in cells and animals, can lead to inter-experimental variation in regards to the size of primary tumor generated and the number of metastases created within the lung. However, although the absolute number of metastases present within the lungs differed between experiments, the rise in metastases following surgical excision of the primary tumor occurred consistently, as did the inhibition of surgery’s effect on metastasis with peri-operative treatment with gefitinib. Additionally, our study is limited by the fact that it was conducted with only one OS cell line and within one mouse strain, and it is possible that the contribution of macrophages to surgery-accelerated metastasis may differ between OS cell lines and in mice of different genetic backgrounds. This will be focus of future studies. We note however, that macrophages have been implicated in surgery-accelerated metastasis in several different tumor types, including breast cancer and melanoma [11, 18].

In order to examine the distinct effects that surgical wounding and removal of the primary tumor may have on surgery-accelerated metastasis, we included a group in our experiments where the contralateral non-tumor-bearing limb was amputated. This subjected animals to the equivalent surgical stress of an amputation, while leaving the primary tumor in place. Interestingly, amputation of the non-tumor bearing limb did not result in the same increase in metastatic nodules as amputation of the primary tumor bearing-limb. In the acute post-operative period, surgical intervention regardless of removal of the primary tumor resulted in a shift toward a pro-tumor macrophage phenotype within the metastatic niche. We hypothesize that the magnitude of the surgical insult incurred by amputation is so significant that it may mask small differences in macrophage phenotype between the two groups. Long-term however, macrophage phenotype in the contralateral amputation group returned to near that of non-operated controls, while those animals that underwent surgical removal of their primary tumor showed a persistently altered pro-tumor pulmonary macrophage phenotype. This suggests that enhanced metastatic outgrowth and persistent tumor-supportive macrophage phenotype within the metastatic niche is specifically related to removal of the primary tumor, and is not solely dependent on factors intrinsic to surgical stress.

As reviewed by Chiarella et al. [37], the ability of the primary tumor to exert a controlling and inhibitory effect on the growth of distant metastases is known as concomitant tumor resistance, and may be a contributory mechanism in surgery-accelerated metastasis. It has been postulated that the primary tumor can inhibit distant metastases either directly through the production of anti-angiogenic and anti-proliferative substances or indirectly by generating an anti-tumor immune response that is capable of inhibiting small micrometastases [37]. Concomitant tumor resistance has been demonstrated in a number of tumor types including sarcomas. In mouse models of both melanoma and sarcoma, Schatten et al. [38] demonstrated that surgical resection of the primary tumor led to an increase in both the frequency and size of pulmonary metastases compared to the no-surgery group or the group that underwent amputation of the non-tumor bearing limb. Similarly, in a model of osteosarcoma, Tsunemi et al. [39] demonstrated that resection of the primary tumor produced an increase in pulmonary metastasis compared to a sham-surgery group, and the increase in metastasis was associated with a systemic reduction in the angiogenesis inhibitor endostatin, resulting in systemic enhancement of angiogenesis. The mechanism by which the primary tumor in our model of OS modulates macrophages to inhibit metastatic growth while in place is unclear, and will be the focus of future work.

Our work and the work of others suggests that removal of the primary tumor may contribute to surgery-accelerated metastasis. However, other studies demonstrate that surgical wounding itself is sufficient to provoke metastatic out growth. Krall et al. successfully create an experimental model of breast cancer where primary tumor resection and surgical wounding are uncoupled. In that report, surgical wounding itself was able to generate a systemic response that led to the outgrowth of tumors at distant sites [11]. Similarly, Hobson et al. [40] demonstrated the acute inflammation generated by biopsy alone was sufficient to increase the frequency of pulmonary metastases in a mouse model of breast cancer. We demonstrate that removal of the primary tumor is needed for surgery-accelerated metastasis in OS; however, these studies underscore the complexity of the mechanisms contributing to surgery-accelerated metastasis. Clinically, tumor resection and surgical wounding occur jointly, however separating these two processes experimentally aids in providing important mechanistic insights.

Although there is a body of work, including this report, that surgical removal of the primary tumor is capable of generating systemic effects that result in accelerated metastasis, we certainly do not suggest that surgical resection be abandoned in any way. To the contrary, our findings demonstrate the importance of continued study in this area to further understand the mechanisms behind surgery-accelerated metastasis. Understanding these mechanisms will allow for the development of novel treatment approaches that will serve to further enhance the benefits of surgery. The findings presented here provide rationale for future work on the mechanisms that govern the pro-tumor macrophage phenotypic alterations that occur after surgical resection and how those alterations contribute to enhanced metastasis. Importantly, this study identifies gefitinib, an already FDA-approved medication, as a potential surgical adjunct to mitigate the unwanted consequences of surgery, thereby enhancing its benefits.

## Conclusions

In conclusion, this study provides novel insights into the mechanisms of surgery-accelerated metastasis in OS. We have demonstrated that surgical excision of the primary tumor leads to a significant increase in pulmonary metastatic nodules and overall metastatic burden. We have shown that this increase in post-surgical metastasis is associated with a persistent phenotypic shift in macrophages within the metastatic niche to a more pro-tumor state that is specific to removal of the primary tumor. Peri-operative treatment with gefitinib resulted in reversal of the pro-tumor macrophage phenotype to a more anti-tumor state, which was associated with a significant reduction in metastases after surgery. Surgery coupled with gefitinib as a maintenance therapy resulted in improved overall survival in this model. Future studies will focus on the downstream mechanisms by which pro-tumor macrophages promote surgery-accelerated metastasis and will investigate macrophage modulation as a therapeutic approach to mitigate the systemic tumor-promoting effects of surgery.

## Supplementary information

**Additional file 1.** Tumor bearing mice succumb with less metastatic burden.

## Data Availability

All data generated or analyzed during this study are included in this published article.

## Abbreviations

OS: Osteosarcoma
TAM: Tumor-associated macrophage
MAM: Metastasis-associated macrophage
RIPK2: Receptor interacting protein kinase 2
MHCII: Major Histocompatibility Class II
ANOVA: Analysis of variance
