# Supplementary material for: Pharmacological prevention of surgery-accelerated metastasis in an animal model of osteosarcoma
Source: J Transl Med. 2020 Apr 30;18:183. doi: 10.1186/s12967-020-02348-2 (PMC7193344; doi:10.1186/s12967-020-02348-2)
Supplement: Supplementary file 1 — Additional file 1. Tumor bearing mice succumb with less metastatic burden. [file 12967_2020_2348_MOESM1_ESM.pdf]

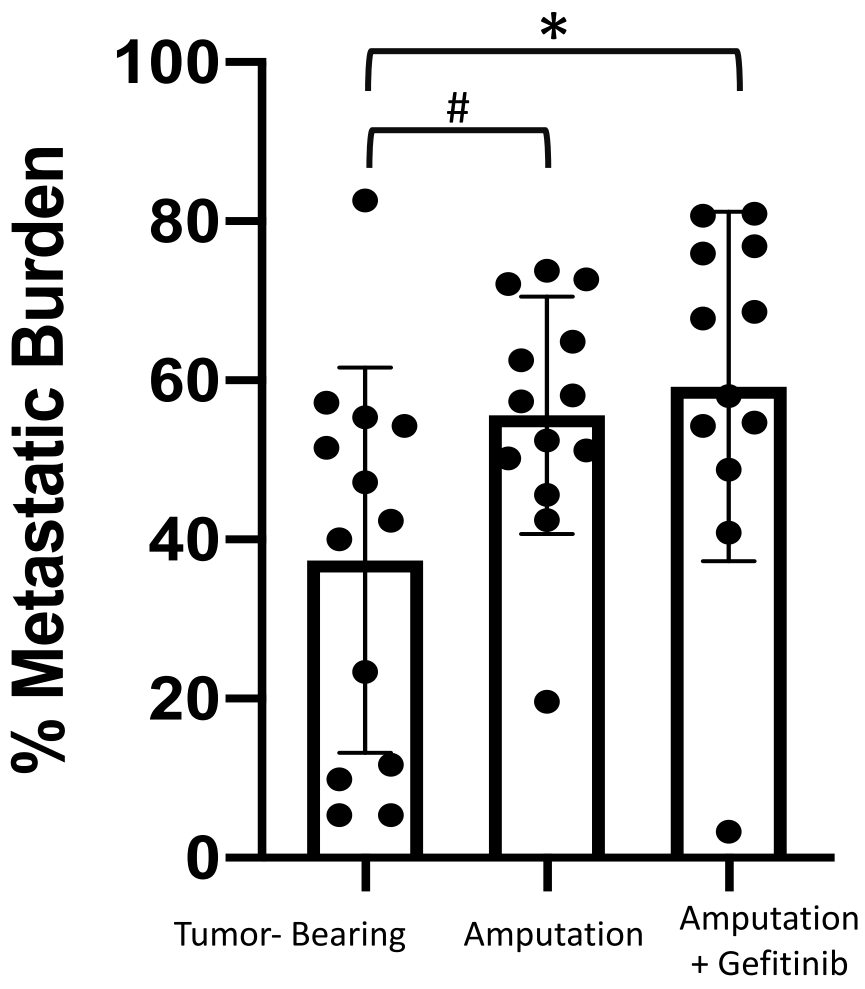

Tumor bearing mice succumb with less metastatic burden

BALB/c mice were implanted with $3x10^5$ cells in the left tibia (n=13-15 mice/group). Mice treated as described in Fig 4. and sacrificed upon meeting euthanasia criteria as previously described. Metastatic burden calculated by measuring the total area of metastatic foci divided by the total area of the lung section from lung on H&E stained lung sections. Data compared by one-way ANOVA with post-hoc Dunnett's multiple comparison test, p=0.26. #p=0.058, *p<0.05
